# Language Dominance and Sociolinguistic Experience Are Related to Language Control and Domain-General Monitoring Control: An Investigation in Bilinguals Who Live in a Minority/Majority Sociolinguistic Setting

**DOI:** 10.3389/fpsyg.2021.594648

**Published:** 2021-08-12

**Authors:** Ruilin Wu, Esli Struys

**Affiliations:** ^1^Centre for Linguistics, Department of Linguistics and Literary Studies, Vrije Universiteit Brussel, Brussels, Belgium; ^2^Centre for Neurosciences, Vrije Universiteit Brussel, Brussels, Belgium

**Keywords:** language dominance, language switching, minority language, language control, cognitive control, language exposure

## Abstract

Bilingual language control in production tasks with language switches is supposed to be linked to domain-general cognitive control. In the present study, we investigated the role of language dominance, measured on a continuous scale, in the relationship between measures of language control elicited through language switching in a picture naming task and non-linguistic cognitive control induced by stimulus-response interference in a Simon task. In our sample of bilinguals who speak both a minority and majority language (language pair of Uyghur-Chinese), the results showed that as bilinguals were more L2-dominant, a pattern of reversed asymmetry switch costs in language control, i.e., larger L2 than L1 switch costs, was observed. Furthermore, the findings showed that recent exposure to the L1 minority language was associated with the change in language switch costs in terms of both response latencies and accuracy rates. This suggests a role for sociolinguistic context in bilingual language control. Concerning cross-domain generality, globally sustained language control was found to be correlated with domain-general monitoring control in response latencies for all bilingual participants. It lends support to the idea that bilinguals tap into monitoring control in the context of language switching. Additionally, the cross-domain overlap was found between two non-equivalent measures (global language control vs. cognitive inhibitory control) in response latencies, specifically for L1-dominant bilinguals. This suggests that language dominance may have an impact on cross-domain generality in language-switching processes.

## Introduction

Bilinguals commonly experience the practice of producing oral utterances in one language and then switching into the other language. This ability of bilingual language control to select the intended language and to inhibit the non-intended language has aroused the interest of researchers (e.g., Green, 1998; Costa and Santesteban, 2004; Abutalebi and Green, 2008; Kroll et al., 2008; Verhoef et al., 2009; Linck et al., 2012; Fink and Goldrick, 2015; Mosca and de Bot, 2017). The landmark study by Meuter and Allport (1999) initially explored bilingual performance through a language-switching naming task. Bilinguals completed both repetition and switch trials in one task by naming single-digit numbers in the same language as in the previous trial (e.g., L1 to L1) or after changing to the other language (e.g., L1 to L2) with reference to a cue. It was found that responses to the switch trials for both languages took longer than to the non-switch trials, but the switch cost for each language differed depending on the relative language strength, with higher switch costs for the dominant than for the non-dominant language.

This empirical finding of dominance-related asymmetric switch costs, as first described by Meuter and Allport (1999), is consistent with the predictions from the inhibitory control (IC) model (Green, 1998). The IC model proposes that the mechanisms underlying bilingual speech production rely on inhibition applied to the non-target language to select the intended language. Specifically, producing speech in the target language is executed through its corresponding language schema, where the activated lexical representations from the non-appropriate language are inhibited through language tags. In the context of switching between two imbalanced languages with one stronger language (L1) and one weaker language (L2), naming in L2 entails a large inhibition of L1, whereas L1 naming invokes only a small inhibition of L2. When switching from L2 to L1, the preceding inhibition of L1 lexical representations has a greater effect on reactivating L1 in subsequent naming. Referring back to the findings of the language switching task (Meuter and Allport, 1999), larger switch costs from the non-dominant L2 to the dominant L1 (hereafter referred to as L1 switch costs) than those from L1 to L2 (hereafter shortened to L2 switch costs) would demonstrate, according to the IC model, that the process of language switching control taps into domain-general inhibition control. It is noteworthy that the pattern of larger L1 switch costs during bilingual production may not be identically present in the modality of bilingual comprehension, because there is some controversy about the direction of switch costs in bilingual comprehension. In the study of language switching in reading, it was found that inhibitory control was recruited during the switch from L1 to L2 reading, while there was no suppression during the switch from L2 to L1 (Bosma and Pablos, 2020). Similarly, another study showed that the presence of switch costs exclusively existed in the switching into L2 reading, not in the reversed direction (Bultena et al., 2015). However, the study by Litcofsky and Van Hell (2017) showed the presence of switch costs in both switching directions and larger costs when switching into the dominant language, which shared similarities with the switching pattern in bilingual production.

### Language Dominance Moderating the Domain-Generality of Language Control

The assumption that the inhibition underlying control over language production is proportionally modulated through the relative proficiency level of each language has been progressively explored through follow-up studies involving different means (Bobb and Wodniecka, 2013). Some studies using a similar unpredictable language switching task have replicated the effect of language proficiency in that switch cost patterns are different between L1-dominant bilinguals (asymmetrical switch costs that are higher in L1) and highly proficient, balanced bilinguals (symmetrical switch costs; Costa and Santesteban, 2004; Finkbeiner et al., 2006; Philipp et al., 2007; Schwieter and Sunderman, 2008; Macizo et al., 2012; Filippi et al., 2014; Fink and Goldrick, 2015; Reynolds et al., 2016). However, there are also inconsistent findings in studies that found symmetrical switch costs in unbalanced bilinguals (Christoffels et al., 2007; Verhoef et al., 2009; Prior and Gollan, 2011; Declerck et al., 2012; Martin et al., 2013) and in balanced bilinguals for languages with a contrast in the relative strength (e.g., strong second language and weak third language, L2-L3; Costa and Santesteban, 2004; Costa et al., 2006), suggesting that either inhibitory control may not be the exclusive source underlying language control or different constituents of domain-general inhibitory control integrate in switch costs (Wang et al., 2009; Bobb and Wodniecka, 2013). Another remarkable finding in the literature is that many studies on voluntary language switching revealed no differences in switch cost patterns between each bilingual dominance group, in that dominant bilinguals shared symmetrical switch costs similar to balanced bilinguals (Gollan and Ferreira, 2009; Gross and Kaushanskaya, 2015); however, as an exception, Gollan et al. (2014) reported a marginal asymmetry in switch costs for unbalanced bilinguals.

The absence of asymmetrical switch costs does not necessarily imply a lack of inhibitory control, because in some studies, symmetry in switch costs was found alongside the dominance reversed language effect (a slower naming for the dominant language compared to the non-dominant language in both repeat and switch trials) and this pattern was found for balanced bilinguals in an unpredictable switch task (Costa and Santesteban, 2004) as well as for unbalanced bilinguals in a predictable and free switch task (Gollan and Ferreira, 2009). It has been suggested that balanced bilinguals who are highly proficient in the less dominant language (L2) apply a weak but consistent inhibition to the slightly dominant language (L1) across switch and non-switch trials (Gollan and Ferreira, 2009; Gollan et al., 2014). That leads to a generally slower naming in the dominant language than in the less dominant one. In other words, the combination of symmetrical switch costs (i.e., equal costs between switch and non-switch trials for each language within the mixed-language block, viewed as an index of local inhibitory control) and a dominance reversed language mixing effect (i.e., costs of each language across mixed- and single-language blocks, slower processing of L1 vs. L2 in a mixed-language block, regarded as an index of global proactive control) may be a signal of inhibitory control (Kroll et al., 2008; Bobb and Wodniecka, 2013). It should be noted that there is a difference between switch costs and mixing costs, with the former referring to the speed or accuracy difference between switch and repeat trials within a mixed-language condition and the latter referring to the speed or accuracy difference between a mixed-language and a single-language condition. The former is considered to be a measure of local, inhibitory and reactive control, and the latter is a measure of global, monitoring and proactive control (Struys et al., 2019a).

Another methodology that has been used to test domain-generality in bilingual language control concerns testing the direct correlation between individual measures of language switching and domain-general cognitive control. Some results of the studies operating this approach demonstrated an absence of correlation between non-linguistic control measured by conflict resolution tasks and n-2 repetition costs (e.g., contrasts between trials CBA-ABA) of language switching in production (Branzi et al., 2016), or only a limited association between cognitive functioning assessed by non-linguistic task switching and performance on the linguistic switching task (Calabria et al., 2012; Prior and Gollan, 2013; Babcock and Vallesi, 2015; but see Declerck et al., 2017). On the contrary, there are studies reporting cross-domain transfer from inhibitory control to language-switching control (Linck et al., 2012; Liu et al., 2018). However, most of these studies have been customised to exclusively explore one subset of bilinguals (but see Babcock and Vallesi, 2015)—either L1-dominant bilinguals (Liu et al., 2018) or balanced bilinguals (Calabria et al., 2012; Branzi et al., 2016)—and they did not take into account the sociolinguistic context in which language switching was performed, even though this is a crucial factor that might have an impact on individual switching behaviour and reliance on domain-general control.

### Language Control and Dominance in a Minority/Majority Sociolinguistic Context

In bilingual communities featuring frequent language switching, the mixed use of two mental language systems increases the need for control between these two coactivated language networks to produce appropriate switching (Green and Wei, 2014; Green, 2018). For bilinguals in an L1 minority and L2 majority language situation, the dominant (majority) language and the heritage language (minority language spoken by indigenous people or immigrants) constitute an asymmetry in the language status. Such disparity in language sociological status may induce asymmetrical chances of switching in each language direction (Stell, 2015). In a minority and majority language interactional context, the sociolinguistic inclination is that in the process of dense code-switching between minority and majority language (Muysken, 2000; Green and Wei, 2014), the former integrates not only lexical items but also grammatical features from the dominant language, which leads to more frequent switching into the majority than into the minority language (Aalberse et al., 2019; for a case in the languages of Uyghur-Chinese, see Sugar, 2017). However, the insertion of minority language lexical items into the majority language in the process of speaking a majority language is less frequent.

More importantly, this asymmetry in switching between minority and majority languages could imply variations in the involvement of inhibitory control. In a study conducted in the Frisian context of the Netherlands, with Dutch as the majority language at the national level and the Frisian language as a minority language, Bosma and Blom (2019) found a selective link between inhibitory control and switching into the minority language but not into the majority language. It has been proposed that different modes of cognitive control underlie varying degrees of switching (Green and Wei, 2014; Green, 2018). Muysken (2000) identifies three different types of code-switching: alternation from uttering phrases or sentences in one language to another, insertion of lexical items from one language into the syntactical framework of another language and dense code-switching with integration of lexical items and syntactical frameworks of two languages. The most critical code-switching experience in relation to cognitive control is dense code-switching in the sense that more frequent usage of this type is related to reduced inhibitory control and enhanced monitoring control (Hofweber et al., 2016). Dense code-switching into the majority language may be governed by the open control mode, where no suppression is applied to either language and both lexical and syntactical representatives of two languages are allowed to be mixed and to be opportunistically selected for speech planning (Green and Wei, 2014; Bosma and Blom, 2019). On the contrary, the coupled control mode is applied to the reversed switching direction, where the majority language alternates with the minority language (Bosma and Blom, 2019), because including lexical items from the minority language is deemed less appropriate when the majority language is spoken, and a certain amount of inhibitory control is present to keep the two language systems separated. Treffers-Daller (2009) suggested that the proportion of inhibitory control may gradually increase as language separation becomes larger or as the degree of language mixing decreases.

The relative sociolinguistic status of bilinguals’ two languages not only modulates the involvement of inhibitory control in the switching direction to each language, but also influences the relative strength of the acquired languages (Montrul, 2015). The external factors that may affect each language accessibility for bilinguals include the degree of language exposure and the rate of language use in different language domains (Silva-Corvalán and Treffers-Daller, 2015). Concerning bilinguals in minority and majority language situations, the societal power of each language differentiates, and the majority language accounts for a relatively large proportion in the domain of socialisation and functions to a great extent as the medium of instruction in compulsory education. This suggests that the discrepancy in strength between minority and majority languages widens under the impact of the lack of symmetrical use across different societal settings.

For minority language speakers, it is, to a great extent, possible that language dominance switches from the early exposure minority language to the late exposure majority language. Some research has pointed out that the increasing strength of the L2 majority language first sizes down the relative dominance of the L1 and then even takes over dominance (Portocarrero et al., 2007; Montrul, 2015). In other words, this dynamic language status is a characteristic of bilinguals with a minority language as their L1, and these speakers can experience a shift from being initially strong in the minority language to being dominant in the majority language.

### The Present Study

The current study focuses on the interaction between domain-specific language control and domain-general cognitive control in bilinguals in a minority and majority language (Uyghur-Chinese) context. Uyghur is a regional minority language spoken by the indigenous ethnic Uyghur people in the Xinjiang Uyghur Autonomous Region of the People’s Republic of China (hereafter shortened as China), where Standard Chinese (hereafter shortened as Chinese) is the official national language. Typologically, the Uyghur language is written in Arabic script and belongs to the Turkic branch of the Altaic language family, whereas the Chinese language is written in a logographic script and is a member of the Sino-Tibetan language family. Of the people in the Xinjiang region, 46.96% speak Uyghur as their mother tongue (Statistical Bureau of Xinjiang, 2019). Out of this group, people living in urban areas, especially young people, are more likely to be bilingual (Awamuslin, 2015). The language use patterns of bilinguals vary depending on distinct contexts. In the family, the Uyghur language dominates communication between family members; the Chinese language is rarely used, but occasionally occurs in conversations between parents and children (Meng, 2013; Han and Johnson, 2020). In the workplace, Uyghur people usually carry on a formal conversation (i.e., meeting or presentation) with colleagues in the Chinese language, and the Uyghur language is used in casual talk or discussion (Meng, 2013). In China, the National Language Committee prescribed the legal and official status of the Uyghur language at the levels of administration, education and social media. In the domain of administration, government documents, seals and signboards of institutions, and official papers are written in both languages, and the two languages are frequently used for oral communication. In terms of education, successful bilingual education programmes exist in which the two languages both function as the medium of instruction (Zhang and Yang, 2021). In the aspect of social media, the mixed use of the Uyghur and Chinese languages is the common method for posting messages and chatting online (Awamuslin, 2015). Recently, several studies have demonstrated that code-switching is increasingly common among Uyghur-Chinese bilinguals (Cabras, 2014; Memtimin, 2016; Sugar, 2017). Code-switching has been found to be either of the alternational type (longer stretches of speech in either language alternate) or of the insertional type, where Chinese lexical items are inserted into Uyghur sentences (Cabras, 2014). The opposite (inserting Uyghur lexical items into Chinese sentences) is far less common. Chinese verbs can also be inserted into Uyghur to form mixed Uyghur verbs (Sugar, 2017). For instance, a mixed verb can consist of a monosyllabic Chinese verb ‘fa’ (send) with the Chinese aspectual marker ‘le’ and a Uyghur auxiliary verb ‘qil-’ (do). Whether the occurrence of mixed verbal compounds can be classified as congruent lexicalisation (dense code-switching) needs to be investigated further, but this type of code-switching is widely used in Uyghur-Chinese communities.

As reviewed in the previous sections, the external linguistic setting contributes greatly to the development of language dominance (de Houwer, 2018), and, in particular, minority language-speaking bilinguals have a gradually higher exposure to the majority language as socialising and schooling level up. Concerning the present study, the proportion of Chinese as the language of instruction gradually increases in the following order: the lowest amount of time is found in ethnic schools, followed by minority and majority joint schools (or bilingual experimental classes), and finally, majority (Chinese) language medium schools (Ma, 2009; Zhang and Yang, 2021). Besides, researchers have argued that language dominance is dynamic and to a great extent depends on what specific modalities (production or recognition) are tested to measure linguistic knowledge (Bahrick et al., 1994; Treffers-Daller, 2015), and balanced bilinguals usually have a tendency towards being slightly more dominant in one language (Hamann et al., 2019).

Therefore, the first research concern of the present study is to investigate whether different degrees of language dominance are associated with different switch cost patterns (larger L1 than L2 switch costs or larger L2 than L1 switch costs) in a bilingual picture naming task with unpredictable switches. Since there is an asymmetry in language control requirements within the context of a minority and a majority language (Bosma and Blom, 2019), together with a possible language dominance shift to the dominant L2 for the minority language-speaking bilinguals (Montrul, 2015), we expect that the language dominance of Uyghur-Chinese bilinguals may vary greatly at the individual level and the dominant language may, to some extent, shift into L2. The variation in language dominance may give rise to a different effect on the relative activation of L1 and L2. Given that the relative costs of L1 and L2 switching depend on language dominance (Meuter and Allport, 1999), the present study hypothesises that, in addition to the switching pattern with larger L1 than L2 switch costs, the asymmetry in language switching might interact with language dominance in the sense that bilinguals with more dominance in L2 show a reversed asymmetry. The study of Bonfieni et al. (2019) suggested that language control is also mediated by individual factors related to language background in that, within a language switching context, an earlier age of L2 acquisition surprisingly predicts slower L2 naming latencies, and a larger amount of L2 exposure relates to smaller L1 switch costs. Given that bilingual language control is modulated by language experience (Bonfieni et al., 2019), we further expect that language control, indicated by the size of language switch costs, correlates with short-term language exposure and the onset age of L2 acquisition.

The second research question is to explore whether the processes of linguistic inhibitory control measured by L1 switch costs or L2 switch costs correlate with domain-general cognitive control and whether the processes of global linguistic control indexed by overall performance on a language task link with cognitive control. Bilingual language control will be examined through the picture naming task, and cognitive control will be tested by using a stimulus-response compatibility task (the Simon task) because the mechanisms of the two tasks share a similarity of inhibitory control over the irrelevant distractor at the response (and not the stimulus) level.

With regard to the present study, we not only investigate inhibitory control represented by the Simon effect, but also examine conflict monitoring control by measuring global performance in a cognitive control task (e.g., Costa et al., 2009; Hilchey and Klein, 2011; Struys et al., 2019b). Monitoring control deserves investigation, because apart from monitoring as a major component in domain-general cognitive control (Miyake et al., 2000), the language switching process is also featured with extensive monitoring, especially in dense code-switching where switching is frequent and unpredictable. Highly proficient bilinguals are likely to switch frequently between either language (Gollan and Ferreira, 2009), which would signify that conflict monitoring is developed to a larger extent in this type of bilingual. Furthermore, previous studies have suggested that in a high monitoring context where the frequency of responding to congruency conditions and incongruency conditions was equal, highly proficient bilinguals responded more efficiently than monolinguals and this difference was more outspoken than in a low monitoring context (Costa et al., 2009).

Thus, we hypothesise that the measure of language control indexed by either L1 switch costs or L2 switch costs interacts with domain-general inhibitory control measured by the Simon effect or with domain-general monitoring control indexed by global performance on the Simon task and that global language performance correlates with domain-general monitoring control. Given that minority language-speaking bilinguals may experience a dominance shift into L2 with increasing exposure to the L2 majority language, we expect that bilinguals with different degrees of language dominance may show different levels of cross-domain generality in control mechanisms. Bilinguals with a dominance shift into L2 may have experienced more demanding language control over two highly competing languages than their counterparts, who have maintained dominance in their native L1 minority language, and, as a result, they may be more efficient in domain-general monitoring.

## Materials and Methods

### Participants

The participants consisted of 89 young bilingual adults (males = 33; females = 56) with the Uyghur minority language as their native language and with the Chinese majority language as their second language. The recruitment of participants took place at a Chinese-medium university in the city of Xi’an, China. All participants were undergraduate students with an average age of 19.55 years (*SD* = 1.37), and they all offered informed consent before participating in the experiment. They reported no impairment in speaking, reading or other language skills. Prior to being admitted to the university, participants were indigenous residents in the cities or prefectures of the Xinjiang region and as the officially recognised regional language, the Uyghur language was learned in both contexts of home and school.

#### Language Profile LEAP-Q

To gain more insight into the participants’ language profiles, all of them filled out an adapted version of the Language Experience and Proficiency Questionnaire (Marian et al., 2007). Generally, the 89 minority-language-speaking bilinguals showed a significant effect of language, *F* (2, 264) = 377.80, *p* < 0.001, $\eta_{p}^{2}$ = 0.741, on the proficiency self-evaluation, in that the mean score of the L1 Uyghur (*M* = 8.97, *SD* = 1.18) was higher than the L2 Chinese (*M* = 7.83, *SD* = 1.33). The preference for language use was significantly different between the two languages, *F* (2, 264) = 271.46, *p* < 0.001, $\eta_{p}^{2}$ = 0.673, with the average preference for using L1 (*M* = 49.30%, *SD* = 15.90) being larger than L2 (*M* = 43.80%, *SD* = 14.00). There was also a significant difference, *F* (2, 264) = 253.74, *p* < 0.001, $\eta_{p}^{2}$ = 0.658, in the frequency of language exposure in that the L1 Uyghur (*M* = 48.66%, *SD* = 14.53) was more exposed to it than the L2 Chinese (*M* = 40.51%, *SD* = 12.64).

Self-evaluated language proficiency and language use preference (see Table 1) were taken into consideration as indices to assess the overall relative language strength of Uyghur (L1) and Chinese (L2). The reason for including self-reported preference in the calculation of language dominance is that this factor was suggested by previous studies as being part of the individual differences that represented bilingual language dominance (Silva-Corvalán and Treffers-Daller, 2015; Caffarra et al., 2016). Our dominance measure shares similarities with the four criteria (i.e., language history, use, proficiency and attitude) that are part of the Bilingual Language Profile (BLP; Birdsong et al., 2012), a standardised language dominance profile measure. The element of language use preference in our measure is an index for personal language attitude towards one language in actual use, which can be viewed as an adaptation of language use and attitude from the BLP. The language proficiency criterion in the BLP was adopted in our measure to represent the participants’ language skills as well. Our measure only excludes the dimension of language history because the target bilinguals acquire the same L1 and L2 (Uyghur and Chinese languages) from the same region and the homogeneity of bilinguals has limited the variation in the dimension of language history. Therefore, it is a reliable approach to use language proficiency and language use preference as the dominance measure. Language proficiency was self-rated on an 11-point scale from the score of 0 to 10 for each literacy skill and for each language. Language use preference was scaled in percentages to indicate the use frequency of L1, L2 and L3 in the following scenarios: reading a book, having a conversation and writing a letter. The sum of the preference percentages for different languages in each scenario should equal 100%. For instance, when reading a book, a bilingual might prefer to read 55% of the time in L1, 35% of the time in L2 and the remaining 10% in L3. Given that both language proficiency and language use preference are the two dimensions that we used to evaluate language dominance, the scale had to be unified by transforming the language use percentage into a score on the same scale as proficiency. In the first step, the three proficiency scores (L1, L2 and L3) for each literacy skill were added. For instance, if one participant evaluates the score of his/her reading skill as 10 for L1, 8 for L2 and 5 for L3, the sum score for reading would be 23. In the second step, the overall score for each literacy skill was multiplied by the percentage of language use preference, yielding a separate score for each language. For instance, in case the same individual reported the following language use preferences, L1 (55%), L2 (35%) and L3 (10%), when reading a book, these percentages of language use preference in the reading scenario were transformed into scores of 12.65 (23^*^0.55) for L1, 8.05 (23^*^0.35) for L2 and 2.3 for L3 (23^*^0.10). Thus, based on this method, there were three transformed scores for each language that indicated the language use preference for reading books, engaging in conversation and writing letters. Additionally, four proficiency scores (two productive and two receptive skills) existed for each language. In total, each participant was assigned seven scores for each language. The strength of each language could be represented by a composite score, that is, the sum of all seven scores. To measure each participant’s language dominance, the first step was to obtain an index of dominance by subtracting the composite score for Chinese from the score for Uyghur. The second step was to convert the index of dominance into a *z*-score, which was used as the final dominance score. The above measurement of language dominance followed the operationalisation proposed in a previous study by Treffers-Daller and Korybski (2015). In the present study, the final dominance score (*z*-score) varied from −2.37 to 2.59, with a mean of 0. As a continuous scale, the closer to or higher than +1 the final dominance score is, the more L1-dominant the bilingual is; the closer to or lower than −1 it is, the more L2-dominant the bilingual is.

**Table 1:** Mean scores and standard deviations (in parenthesis) for language experience background information of bilingual participants.

	Uyghur-Chinese bilinguals (*n* = 89)
Age	19.55 (1.37)
Male/Female	33/56
IQ	46.22 (5.18)
L1 recent exposure^1^	48.66% (14.53)
L2 recent exposure	40.51% (12.64)
Age of L2 acquisition	6.10 (2.23)
L1-Uyghur proficiency^2^	8.97 (1.18)
L2-Chinese proficiency	7.83 (1.33)
L1-Uyghur use preference (in %)	49.30% (15.90)
L2-Chinese use preference (in %)	43.80% (14.00)
L1-Uyghur strength^3^ (composite score)	56.20 (12.40)
L2-Chinese strength (composite score)	49.90 (12.30)
Index of dominance^4^	6.34 (19.57)
Final dominance score (*z*-score of index of dominance)^5^	0 (1)

1 *Recent language exposure was a self-assessed score in percentages. The sum of L1 and L2 exposure was not 100% due to an additional language reported in the LEAP-Q*,

2 *Language proficiency was a self-assessed score ranging on an 11-point scale from 0 (low proficient) to 10 (high proficient)*,

3 *Language strength indicates the sum of three self-rated scores for language proficiency and four self-rated scores for language use preference (transformed)*,

4 *Index of dominance was calculated by subtracting L2-Chinese strength from L1-Uyghur strength and*

5 *Final dominance score ranged from −2.37 to 2.59, and the mean was approximately equal to 0*.

### Materials and Tasks

#### Picture Naming Task

The picture naming task involved a mix of repeat and switch trials and was adapted from the version used in a previous study by Linck et al. (2012). Eleven pictures with non-coloured line drawings were selected from the standardised picture set provided by Snodgrass and Vanderwart (1980). The selection of the 11 standardised pictures was based on the criteria of agreement in naming, the association between the picture and the mental image of the object, and the word frequency. To be specific, descriptive statistics for the criterion of naming agreement of the selected pictures (*M* = 97.55%, *SD* = 2.81) were retrieved from the study of Weekes et al. (2007), and the relevant data on image agreement (*M* = 4.39, *SD* = 0.19) and the word frequency (*M* = 3.51, *SD* = 0.71), which were based on the subjective evaluation indicated by the scale from 1 to 5, were accessible in the research of Liu et al. (2011). Given that the Uyghur language is comparatively underexplored, the picture selection is mainly based on the available criteria statistics data concerning the Chinese language. Each picture in an individual presentation appeared 12 times, and 132 pictures (trials) were generated in total. Each trial contained one picture as the stimulus, and 12 lists were used to present all 132 trials. Each list varied in length depending on the number of trials (pictures) included. To realise the pseudo-randomised distribution across the two languages for presenting 40 switch trials and 80 non-switch trials, we allowed for differences in the length of each list. The minimum length of the picture list was 5, and the maximum length extended to 17 pictures. At the end of each list, an asterisk appeared to indicate the short interval (500 ms) preceding the presentation of the next list. The first picture presented in the list was taken as the baseline to determine whether the following trial could be counted as a switch or a non-switch trial. In other words, the first picture did not belong to either trial type, so the 12 first pictures in each of the 12 lists should be subtracted from the total number of 132 trials when calculating the switch or non-switch conditions. In total, the distribution was 40 switch trials and 80 non-switch trials, and L1 and L2 were, respectively, assigned the same proportion of switch (20 trials per language) and non-switch trials (40 trials per language).

The specific parameter setting for each trial is described as follows. The fixation sign (+; 1,000 ms) was presented at the beginning of the list and then followed by a blank interval (500 ms). Then, the cue (500 ms) to indicate the naming language was presented prior to the occurrence of the picture. The cue was the equivalent word of ‘speak’ written in either Uyghur or Chinese. Showing words as cues is indeed a less common way to indicate the relevant language for naming. However, non-linguistic colour cues may increase the demands on working memory required to bear in mind the rules about the correspondence between colour and language. Thus, showing words only minimally elicits language comprehension processes, but at the same time minimises the confound effect of working memory demands on naming results. The picture was presented until the participant gave a spoken response. After the response, the picture was no longer present on the screen and instead the sign of a dot would appear to inform the participants that the naming was recorded; if no naming response was detected within 4,000 ms, it would pass on to the next trial.

#### Raven’s Progressive Matrices

The standard version of Raven’s Progressive Matrices (Raven, 1938) was administered to control for the factor of the IQ of each participant because this element has been suggested by previous research as being closely related to the ability of cognitive control (Arffa, 2007). This non-linguistic version of the IQ test aimed at assessing logic and deductive reasoning, and it was composed of five blocks of tests from level A to level E in order of increasing difficulty. Each block consisted of 12 matrices; 60 matrices were tested in total. The correct response to each matrix was counted as 1 point, with up to 60 points as the full score. Of the participants, 89 were on average with an IQ score of 46.22 (*SD* = 5.18).

#### Simon Task

Coloured squares in red or blue were presented individually as the stimuli of the Simon task (Simon and Rudell, 1967). The red or blue square could be placed on the left side, the central position or the right side of the screen. The required response was based on the rule of recognising the colour of the stimulus instead of its location. The response to the red square was matched to pressing the left key (key A on the keyboard), whereas the blue square was responded to by pressing the right key (key L on the keyboard). On a congruent trial, the location of the stimulus matched with the correspondent response press key; on a neutral trial, either coloured square was presented in the centre; and on an incongruent trial, the location of the stimulus was on the opposite side of the relevant response.

In total, 126 trials were equally distributed across each trial type with 42 congruent, 42 neutral and 42 incongruent trials. The red or blue squares were equally presented with 21 trials per coloured square for each trial condition. The specific process for presentation started with a fixation (500 ms) preceding a blank interval (250 ms), followed by the stimulus. The stimulus was presented on a white screen for a maximum length of 2,500 ms, and it would disappear when a response was given by the participants. All stimuli appeared in random order.

#### Procedure

The set of experiments included one language task, one cognitive control task and one non-linguistic intelligence test. All tasks were administered separately for each individual participant. For both linguistic and non-linguistic tasks, the experiment was conducted in a studio with sound insulation, and for the picture naming task, all naming was collected through a microphone and recorded and timed by a MacBook Pro laptop with a 15.4-inch screen. The participants completed the experiments in the following sequence: the language task, the Simon task and then Raven’s Progressive Matrices. Prior to the actual experiment, a small block of practice trials was administered to the participants to inform them of the task requirements. The presentation of the stimuli was designed using HTML5 and JavaScript programming languages, and all tasks were administered through the Google Chrome browser. All collected data were recorded in the MySQL database (Axmark and Widenius, 2015), a software using structured query language to access, process and manage a large amount of data. The timing of the spoken response was collected *via* the application programming interface (API) of Web Speech to enable the web browser to recognise the speech input. The technique of API innate in the Google Chrome browser is flexible and accurate for precisely recognising the timing of a spoken response.

## Results

The analyses of accuracy rates (score 1 for correct response and score 0 for incorrect naming or no response) were conducted with mixed logistic regression modelling (Jaeger, 2008), and response times (RTs) were analysed with linear mixed-effects regression models. Outliers larger or smaller than 2.5 standard deviations from the mean response time of correct trials were removed from further analyses of the picture naming task and the Simon task. In the picture naming task, 330 out of 11,236 trials (2.9% data) were removed; in the Simon task, 271 out of 10,844 trials (2.5% data) were eliminated.

The packages of lme4 (version 1.1–21; Bates et al., 2015) and the lmerTest (version 3.1–1; Kuznetsova et al., 2017) in the statistical software R, version 3.6.3 (R Core Team, 2020), were used to build models. The continuous variable of Language Dominance was a set of standardised *z*-scores centred at 0. The categorical fixed factors of Language and Switch Type were centred and contrast-coded with the sum coding (e.g., Switch Type: −0.5 = non-switch trials vs. 0.5 = switch trials), and then, the main effects of the categorical predictors in the model were tested. Assigning values of −0.5 and +0.5 is a variant of sum coding, and it provides an easier way to interpret the results of the model (Schad et al., 2020). If an interaction effect was found to be significant, follow-up regression analyses on each level of the combinations of related factors were conducted to examine the interaction (Gollan and Goldrick, 2016). The significance (Chi-square statistics) of the model fixed effects was analysed through a model comparison based on the Type III sum of squares analysis (Zahn, 2010).

### Picture Naming Task

The logistic regression model fitted to the individual accuracy rates included two categorical fixed factors of Switch Type (sum coding: switch = 0.5, non-switch = −0.5), and Language (L1-Uyghur = 0.5, L2-Chinese = −0.5). One continuous fixed factor of Language Dominance was included, which was composed of standardised scores, i.e., *z*-scores ranging from −2.37 to 2.59 with a mean of 0. Scores closer to or above +1 meant higher dominance in L1-Uyghur, while scores closer to or below −1 represented higher dominance in L2-Chinese. The model was maximally fit with intercept random effects at the subject and picture item levels (Barr et al., 2013). The output of this logistic regression model is summarised in Table 2. The descriptive statistics are reported in Table 3. The findings showed a main effect of Switch Type [*β* = −0.91, *SE β* = 0.11, *χ^2^* (1) = 73.33, *p <* 0.001], and bilingual individuals produced a higher ratio of correct answers on the repeat trials (*M* = 98.20, 95% CI = 97.60–98.60%) than on the switch trials (*M* = 95.60, 95% CI = 94.30–96.60%). There was no main effect of Language [*β* = 0.05, *SE β* = 0.11, *χ^2^* (1) = 0.24, *p* = 0.622] or of Language Dominance (*β* = −0.03, *SE β* = 0.10, *χ^2^* (1) = 0.07, *p* = 0.794). Language Dominance did not interact with Language [*β* = −0.03, *SE β* = 0.10, *χ^2^* (1) = 0.07, *p* = 0.798]. Neither was there any interaction between Switch Type and Language Dominance [*β* = −0.12, *SE β* = 0.10, *χ^2^* (1) = 1.21, *p* = 0.272].

**Table 2:** Results for logistic mixed-effects model of accuracy probability in the picture naming task.

	Model summary			Model effect significance		
	*β*	*SE β*	*z*	*χ^2^*	*df*	Value of *p*
***Fixed effects***						
(Intercept)	3.53	0.13	27.37	**748.91**	**1**	**< 0.001**
Switch Type	−0.91	0.11	−8.56	**73.33**	**1**	**< 0.001**
Language	0.05	0.11	0.49	0.24	1	0.622
Dominance	−0.03	0.10	−0.26	0.07	1	0.794
Switch Type * Language	0.37	0.21	1.75	3.07	1	0.080
Switch Type * Dominance	−0.12	0.10	−1.10	1.21	1	0.272
Language * Dominance	−0.03	0.10	−0.26	0.07	1	0.798
Switch Type * Language * Dominance	−0.55	0.21	−2.61	**6.82**	**1**	**< 0.01**

*Dominance stands for Language Dominance. Significant statistics are demonstrated in bold*.

**Table 3:** Mean accuracy rates (%), mean correct response times (ms) and the 95% confidence intervals (CI) from the lower level to the upper level for the picture naming task by Switch Type and Language.

	Accuracy rates		Response times	
	Mean	95% CI	Mean	95% CI
Non-switch Uyghur	98.00	97.30–98.60	1,078	1,050–1,106
Switch Uyghur	96.10	94.70–97.10	1,163	1,135–1,192
Non-switch Chinese	98.30	97.70–98.70	1,080	1,052–1,108
Switch Chinese	95.00	93.40–96.30	1,171	1,142–1,199

There was a marginal significance of the interaction effect between Switch Type and Language [*β* = 0.37, *SE β* = 0.21, *χ^2^* (1) = 3.07, *p* = 0.080]. The follow-up regression models at each level of Language showed that there was a slight difference in the size of the switch effect on the Uyghur [*β* = −0.74, *SE β* = 0.15, *χ^2^* (1) = 25.61, *p <* 0.001] and the Chinese [*β* = −1.10, *SE β* = 0.15, *χ^2^* (1) = 56.97, *p <* 0.001] naming accuracy, indicating that the switch effect on naming accuracy in Uyghur (*M_non-switch_* = 98, 95%CI*_non-switch_* = 97.30–98.60%, *M_switch_* = 96.10, 95%CI*_switch_* = 94.70–97.10%) was slightly smaller than that in Chinese (*M_non-switch_* = 98.30, 95%CI*_non-switch_* = 97.70–98.70%, *M_switch_* = 95, 95%CI*_switch_* = 93.40–96.30%).

**Figure 1:**
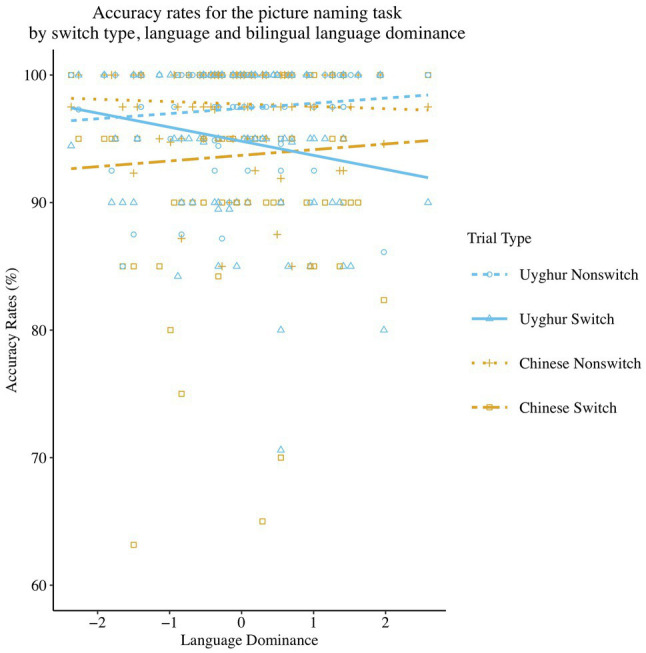
Scatterplot and regression fit lines showing the correlation between Language Dominance and mean accuracy rates at each level of the combinations of Switch Type and Language in the picture naming task.

A significant three-way interaction (see Figure 1) was found between Language Dominance, Language and Switch Type [*β* = −0.55, *SE β* = 0.21, *χ^2^* (1) = 6.82, *p <* 0.01]. The follow-up regression models at each level of the four combinations of factors for Language and Switch Type showed that accuracy rates for the Uyghur language on the switch trials were significantly lower [*β* = −0.22, *SE β* = 0.11, *χ^2^* (1) = 4.24, *p <* 0.05] as participants were more L1-dominant, whereas no significant effect of Language Dominance was found [*β* = 0.17, *SE β* = 0.11, *χ^2^* (1) = 2.66, *p* = 0.103] in accuracy rates on the repeat trials of the Uyghur language, on Chinese naming speed on the non-switch trials [*β* = −0.08, *SE β* = 0.11, *χ^2^* (1) = 0.55, *p* = 0.458] or on the Chinese switch trials [*β* = 0.08, *SE β* = 0.10, *χ^2^* (1) = 0.64, *p* = 0.424].

The RTs analyses in the linear regression model contained the same parameters of fixed and random predictors as the ones in the model for accuracy rates. The output of this regression model is summarised in Table 4. The results showed a main effect of Switch Type [*β* = 88.05, *SE β* = 4.34, *χ^2^* (1) = 411.69, *p <* 0.001], indicating a slower response on switch trials (*M* = 1,167 ms, 95% CI = 1,139 ms-1,195 ms) than on non-switch trials (*M* = 1,079 ms, 95% CI = 1,052 ms-1,106 ms). There was no main effect of Language [*β* = −4.75, *SE β* = 4.34, *χ^2^* (1) = 1.20, *p* = 0.274]. No main effect of Language Dominance was found [*β* = −8.23, *SE β* =12.83, *χ^2^* (1) = 0.41, *p* = 0.521].

**Table 4:** Results for linear mixed-effects model of response times in picture naming task.

	Model summary			Model effect significance		
	*β*	*SE β*	*t*	*χ^2^*	*df*	Value of *p*
***Fixed effects***						
(Intercept)	1123.04	13.93	80.59	**6495.22**	**1**	**< 0.001**
Switch Type	88.05	4.34	20.29	**411.69**	**1**	**< 0.001**
Language	−4.75	4.34	−1.09	1.20	1	0.274
Dominance	−8.23	12.83	−0.64	0.41	1	0.521
Switch Type * Language	−4.82	8.68	−0.56	0.31	1	0.578
Switch Type * Dominance	−0.57	4.36	−0.13	0.02	1	0.896
Language * Dominance	−17.58	4.36	−4.03	**16.26**	**1**	**< 0.001**
Switch Type * Language * Dominance	40.06	8.72	4.59	**21.11**	**1**	**< 0.001**

*Dominance stands for Language Dominance. Significant statistics are demonstrated in bold*.

The two-way interaction was significant between Language and Language Dominance [*β* = −17.58, *SE β* = 4.36, *χ^2^* (1) = 16.26, *p <* 0.001]. The follow-up regression models for each language showed that naming in the Uyghur language was significantly faster [*β* = −20.61, *SE β* = 3.39, *χ^2^* (1) = 36.95, *p <* 0.001] when Language Dominance was more towards L1, while there was a marginally significant tendency [*β* = 5.86, *SE β* = 3.37, *χ^2^* (1) = 3.02, *p* = 0.082] towards a slower response speed in the Chinese language as bilinguals were more L1-dominant. There was no significant interaction [*β* = −4.82, *SE β* = 8.68, *χ^2^* (1) = 0.31, *p* = 0.578] between Switch Type and Language, nor was there any interaction [*β* = −0.57, *SE β* = 4.36, *χ^2^* (1) = 0.02, *p* = 0.896] between Switch Type and Language Dominance.

**Figure 2:**
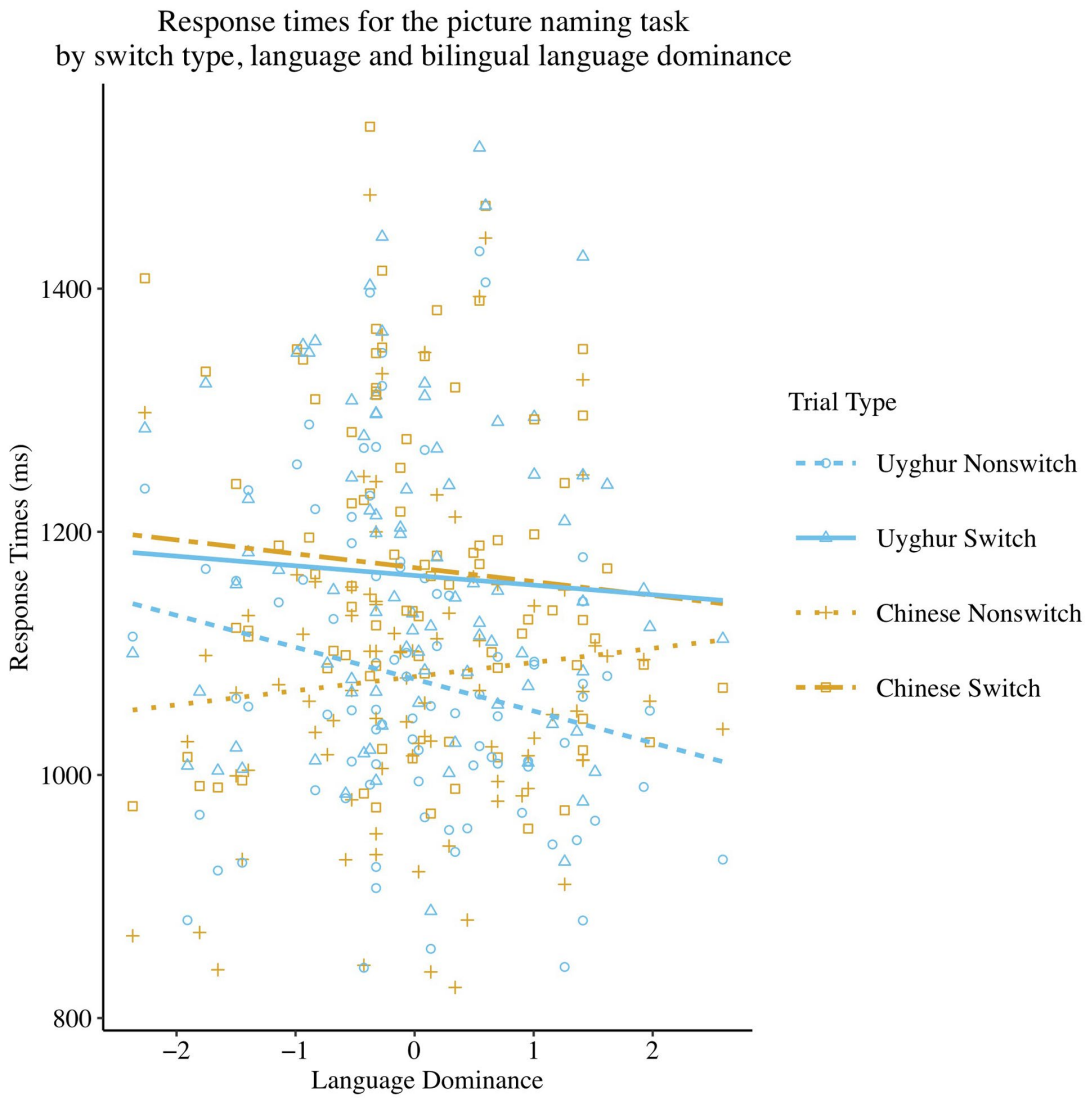
Scatterplot and regression fit lines showing the correlation between Language Dominance and mean response times at each level of the combinations of Switch Type and Language in the picture naming task.

Furthermore, a three-way interaction (see Figure 2) was found to be significant [*β* = 57.32, *SE β* = 19.08, *χ^2^* (1) = 9.03, *p <* 0.01] between Switch Type, Language and Language Dominance (see Table 3 for descriptive statistics). The follow-up regression models at each level of the four combinations of factors for Language and Switch Type showed that the naming latencies were significantly faster [*β* = −26.42, *SE β* = 13.28, *χ^2^* (1) = 3.96, *p <* 0.05] in the Uyghur language on the repeat trials, as Language Dominance was more L1-dominant, while there was no significant effect of Language Dominance on Uyghur naming responses on the switch trials [*β* = −8.60, *SE β* = 13. 81, *χ^2^* (1) = 0.39, *p* = 0.533], on Chinese naming speed on the non-switch trials [*β* = 11.25, *SE β* = 13.95, *χ^2^* (1) = 0.65, *p* = 0.420] or on Chinese switch trials [*β* = −10.82, *SE β* = 13.81, *χ^2^* (1) = 0.61, *p* = 0.433]. It indicated that the Uyghur switch costs were larger for bilinguals more towards the L1-dominance, but the switch effect on Uyghur was smaller for those more dominant in L2. The follow-up exploration of the relationship between the switch costs and language dominance was further conducted in the subsection of correlation analyses.

### Simon Task

The logistic model for the analyses of accuracy rates (for descriptive information, see Table 5) included the fixed factors of Language Dominance and Stimulus Type with three conditions [sum coding: values (0.5, 0) assigned for the congruent trial, (−0.5, −0.5) for the neutral trial and (0, 0.5) for the incongruent trial]. The factor of subjects was fitted as a random effect. Given that the factor of Stimulus Type has three levels, the follow-up pairwise comparison was conducted to indicate the contrast between each level of condition type; therefore, three estimated contrasts are reported here. The main effect of Stimulus Type was found to be significant [*χ^2^* (2) = 70.17, *p <* 0.001], with a significantly higher rate of correct responses for congruent trials (*M* = 98.07, 95% CI = 97.53–98.50%) than for incongruent trials (*M* = 95.29, 95% CI = 94.35–96.08%), *β* = 0.92, *SE β* = 0.13, *z* = 6.90, *p <* 0.001. Accuracy scores on the incongruent trials were significantly lower (*β* = −0.87, *SE β* = 0.13, *z* = −6.64, *p* < 0.001) than on the neutral trials (*M* = 97.98, *95% CI* = 97.42–98.42%), and there was no significant difference (*β* = 0.05, *SE β* = 0.16, *z* = 0.31, *p* = 0.949) between the congruent and the neutral trials. No main effect of Language Dominance was found, nor was there any interaction effect (*p*s > 0.786).

**Table 5:** Mean accuracy rates (%), mean correct response times (ms) and the 95% confidence intervals (CI) from the lower level to the upper level for the Simon task by trial types.

	Accuracy rates		Response times	
	Mean	95% CI	Mean	95% CI
Congruent	98.07	97.53–98.50	678	662–693
Neutral	97.98	97.42–98.42	688	673–704
Incongruent	95.29	94.35–96.08	712	696–727

The regression analyses on the RTs using the same fixed and random factors showed that there was a significant main effect of Stimulus Type [*χ^2^* (2) = 210.82, *p <* 0.001]. The pairwise comparisons indicated a significant Simon effect with faster responses on the congruent trials (*M* = 678 ms, *95% CI* = 662 ms-693 ms) than on the incongruent trials (*M* = 712 ms, 95% CI = 696 ms-727 ms), *β* = −34.10, *SE β* = 2.40, *t* = −14.22, *p* < 0.001. The RTs on the neutral trials (*M* = 688 ms, 95% CI = 673 ms-704 ms) were significantly slower than those on the congruent trials (*β* = −10.70, *SE β* = 2.38, *t* = −4.49, *p* < 0.001), but the responses in the neutral trials were significantly faster than in the incongruent ones, *β* = 23.40, *SE β* = 2.40, *t* = 9.76, *p* < 0.001. There was no main effect of Language Dominance, and no two-way interactions were found (*p*s > 0.129).

### Correlation Analyses

Further correlation analyses were conducted to explore to what extent the switch costs in either language in the picture naming task were correlated with language dominance, and to investigate whether the sociolinguistic factors of recent language exposure and age of L2 acquisition contributed to asymmetrical switch costs and to a change in language dominance. The Pearson correlation analyses were executed, respectively, for the RTs and accuracy rates. The correlation was conducted for all 89 participants among the measures of language dominance scores (the same set of *z*-scores as previously used in the regression model analyses), language exposure, initial age of L2 acquisition, L1 switch costs and L2 switch costs (the difference between switch and non-switch trials at each language) and the overall switch costs (the difference between switch and non-switch trials irrespective of language types). An overview of all correlation coefficients for the RTs and accuracy scores is reported in Tables 6 and 7. A significantly positive correlation was found between L1 switch costs and language dominance in both the RTs [*r* (87) = 0.29, *p <* 0.01] and accuracy [*r* (87) = 0.26, *p <* 0.05] analyses, whereas L2 switch costs were significant in a negative interaction [*r* (87) = −0.33, *p <* 0.01] with language dominance only in terms of the RTs. The increase in L2 switch costs, as well as the decrease in L1 switch costs, correlated with higher L2-dominance, while for higher L1-dominance, it was the other way around. Moreover, the findings showed that lower L1 recent exposure was significantly interrelated to larger naming latencies in L2 switch costs [*r* (87) = −0.24, *p <* 0.05] and with higher error rates under the L2 switch effect [*r* (87) = −0.23, *p <* 0.05]. The correlation analyses revealed that an earlier age of L2 acquisition was significantly associated with lower error rates under the L1 switch effect [*r* (87) = 0.25, *p <* 0.05].

**Table 6:** Bilingual’s Pearson correlation analyses between Language Dominance, recent exposure, initial age of L2 acquisition (AoA L2) and switching performance in the picture naming task in terms of response times (RTs).

	Language Dominance	L1 exposure	L2 exposure	AoA L2	L1 costs RTs	L2 costs RTs	Switch costs RTs
Language dominance	–						
L1 exposure	0.51^****^	–					
L2 exposure	−0.43^****^	−0.86^****^	–				
AoA L2	0.16	0.00	−0.01	–			
L1 costs RTs	0.29^**^	0.08	−0.14	0.00	–		
L2 costs RTs	−0.33^**^	−0.24^*^	0.15	−0.10	0.17	–	
Switch costs RTs	−0.05	−0.11	0.02	−0.07	0.74^****^	0.79^****^	–

*A higher score in Language Dominance means more dominance in L1 (Uyghur). N = 89*.

* *p **<** 0.05*;

** *p **<** 0.01* *and*

**** *p **<** 0.0001*.

**Table 7:** Bilinguals’ Pearson correlation analyses between Language Dominance, recent exposure, initial age of L2 acquisition (AoA L2) and switching performance in the picture naming task in terms of accuracy rates (ACC).

	Language Dominance	L1 exposure	L2 exposure	AoA L2	L1 costs ACC	L2 costs ACC	Switch costs ACC
Language dominance	–						
L1 exposure	0.51^****^	-					
L2 exposure	−0.43^****^	−0.86^****^	–				
AoA L2	0.16	0.00	−0.01	–			
L1 costs ACC	0.26^*^	0.12	−0.15	0.25^*^	–		
L2 costs ACC	−0.08	−0.23^*^	0.21	−0.03	0.06	–	
Switch costs ACC	0.09	−0.10	0.07	0.13	0.65^****^	0.80^****^	–

*A higher score in Language Dominance means more dominance in L1 (Uyghur). N = 89*.

* *p **<** 0.05* *and*

**** *p **<** 0.0001*.

The second examination of the relationship between domain-specific and domain-general control was conducted through a correlation analysis of measures of the language and cognitive control tasks. First, for all 89 participants, the analyses were conducted for the RTs and accuracy rates to correlate the L1 switch costs, L2 switch costs and the overall switch effect (the difference between switch and non-switch trials across the language types), respectively, with inhibitory control abilities indexed by the Simon effect (the difference between congruent and incongruent trials) in the cognitive control task. Another dimension of analysis was aimed at the correlation of the previously mentioned measures in the language task with global performance in the Simon task. The coefficient results of the RTs and accuracy rates are reported in Table 8. A significantly positive correlation [*r* (87) = 0.32, *p <* 0.01] was found between global language control and global monitoring control in terms of response latencies.

**Table 8:** Bilinguals’ (all participants) Pearson correlations between measures of linguistic control in the picture naming task (shortened as PNT) and of cognitive control in the Simon task in terms of response times (RTs) and accuracy rates (ACC).

Measures of linguistic control	Measures of cognitive control	Coefficients for RTs (*n* = 89)	Coefficients for ACC (*n* = 89)
L1 switch costs	Simon effect	0.00	0.01
	Simon monitoring	0.06	0.05
L2 switch costs	Simon effect	0.13	0.03
	Simon monitoring	−0.02	−0.14
Switch costs	Simon effect	0.09	0.03
	Simon monitoring	0.03	−0.08
PNT monitoring	Simon effect	0.10	−0.15
	Simon monitoring	0.32^**^	0.17

** *p **<** 0.01*.

Second, since the findings suggest that language dominance plays an important role in language control, there was a need to take into consideration the effect of language dominance on moderating the relationship between language control and domain-general control. Thus, follow-up separate correlation analyses for L1- and L2-dominant bilinguals were conducted among the same measures of language and cognitive control as used for the overall participants. Of the participants, 42 bilinguals with a language dominance *z*-score above the mean of 0 were grouped into more L1-dominant, while 47 bilinguals with a language dominance score below the mean of 0 were classified as more L2-dominant. A partial correlation was conducted by controlling for IQ for L1-dominant (*M* = 44.81, *SD* = 4.79) and L2-dominant groups (*M* = 47.49, *SD* = 5.24), due to the significant difference in the measure of IQ between each language dominance group, [*t* (87) = −2.51, *p <* 0.05]. The coefficient results of the RTs and accuracy rates for both groups are reported in Table 9. It was found that for both the L2-dominant [*r* (44) = 0.36, *p <* 0.05] and the L1-dominant group [*r* (39) = 0.43, *p <* 0.01], shorter response latencies in global language control significantly correlated to a faster performance in global monitoring control in the cognitive task. For the L1-dominant group, the faster response latencies in global language control also significantly [*r* (39) = 0.31, *p <* 0.05] correlated with a smaller Simon effect.

**Table 9:** Pearson correlations between measures of linguistic control in the picture naming task (shortened as PNT) and of cognitive control measured by the Simon task in terms of response times (RTs) and accuracy rates (ACC), respectively, conducted for L1- and L2-dominant bilinguals with controlling for IQ.

Measures of linguistic control	Measures of cognitive control	Coefficients for L2-dominant bilinguals (*n* = 47)		Coefficients for L1-dominant bilinguals (*n* = 42)	
		RTs	ACC	RTs	ACC
L1 switch costs	Simon effect	−0.16	−0.12	0.12	0.05
	Simon monitoring	−0.10	0.10	0.16	0.04
L2 switch costs	Simon effect	0.10	−0.14	0.27	0.10
	Simon monitoring	0.05	0.00	0.03	−0.24
Switch costs	Simon effect	−0.03	−0.20	0.27	0.10
	Simon monitoring	−0.02	0.06	0.12	−0.15
PNT monitoring	Simon effect	0.01	0.02	0.31^*^	−0.26
	Simon monitoring	0.36^*^	0.16	0.43^**^	0.19

* *p **<** 0.05* *and*

** *p **<** 0.01*.

## Discussion

In this study, Uyghur-Chinese bilinguals with varying degrees of language dominance were presented with a production-based language switching task and a cognitive control task to investigate to what extent differences in language dominance may have an impact on (a) the asymmetry of switch costs in a bilingual picture naming task and (b) the relationship between domain-specific and domain-general control processes.

### L2 Switch Costs Are Asymmetrically Larger Than L1 Switch Costs in Bilinguals With Higher L2 Dominance

A picture naming task was administered to test the spoken language switching performance of Uyghur-Chinese bilinguals who live in a sociolinguistic context with a minority and majority language. We predicted that language dominance might have an impact on the pattern of switch costs (e.g., Costa and Santesteban, 2004). Specifically, the expectation for bilinguals with more L1-dominance was that they would demonstrate asymmetrical switch costs in response speed (larger costs for switching to dominant L1 than to non-dominant L2). In line with studies on the unpredictable language-switching paradigm (see Bobb and Wodniecka, 2013), the findings of our study show that bilinguals with more L1-dominance experience greater costs to switch into L1 (Uyghur), but smaller costs to switch into L2 (Chinese), in terms of speed in the language production process. Moreover, it was further expected that the asymmetry of L1 and L2 switch costs would be reversed for bilinguals who are more dominant in L2. Unlike previous studies splitting bilinguals into categorical groups (e.g., Schwieter and Sunderman, 2008; Fink and Goldrick, 2015; Reynolds et al., 2016), our study with a continuous scale of measuring language dominance reveals that the size of L2 switch costs is asymmetrically larger than that of L1 switch costs for bilinguals with higher dominance in L2. Particularly, for accuracy, it shows that a higher dominance in L2 is associated with a reduced cost of switching into L1; in terms of speed, greater dominance in L2 relates not only to higher costs for switching into L2, but also to smaller switch costs in the other direction. Our present language production study suggests for the first time that higher L2 dominance in highly proficient bilinguals is related to lower costs of switching into the dominant L1 language.

Sociolinguistic factors, such as short-term language exposure and the initial age of L2 acquisition, are taken into consideration to further investigate whether these variables contribute to the dynamics of dominance and the asymmetry of switch costs in the language control process. In the dimension of response speed and accuracy, the results reveal that the length of recent language exposure comes into play to interact with bilingual language dominance and affects the relative size of switch costs. Particularly, the shorter length of L1 exposure and the longer L2 exposure are related to a higher dominance in L2 of bilinguals, and higher dominance in L2 relates to smaller switch costs towards L1. This result extends previous research on the relationship between language dominance and the size of switch costs in the bottom-up comprehension process to the top-down production process (Bultena et al., 2015).

Moreover, in line with the previous finding that exposure length to one language relates to the size of switch costs in the opposite language (Bonfieni et al., 2019), the present study reveals that bilinguals’ L1 recent exposure is related to L2 switch costs in terms of both response speed and accuracy rates. Specifically, bilinguals with a reduced amount of recent L1 exposure experience an increase in L2 naming latencies and in L2 error rates caused by a language switch. That is, the level of L1 exposure affects the degree of suppression of L2 access in the context of a less-exposed L1. This finding further implies that the level of recent language exposure is critical to moderate language control in bilinguals.

The factor of age of L2 acquisition plays a limited role in its contribution to linguistic control and the link to the dynamic feature of proficiency. The positive effect of the initial age of L2 acquisition on L1 switch costs was found only in terms of accuracy rates. This finding is compatible with prior research suggesting that control over bilingual linguistic networks relates to the factor of L2 age (Sulpizio et al., 2020), but it seems to be limited to accuracy rates and does not include speed of processing. Our findings suggest that bilinguals with an earlier age of L2 acquisition have lower error rates when switching towards L1.

These connections show that the sociolinguistic environment with a predominant majority language and a non-dominant minority language influences language access in both languages and has a further effect on control over these two language systems. Moreover, the findings suggest that bilinguals are responsive to subtle individual variations in the interaction of minority and majority languages, even within the same sociolinguistic context, and that language control processes are moderated by language dominance, which is an adaptive feature of an individual’s language background that is responsive to short-term changes in language use and exposure to multiple languages in a heterogeneous sociolinguistic environment. This finding provides additional support for the adaptive control hypothesis (Green and Abutalebi, 2013), which assumes a high adaptability of language control processes in response to the interactional setting in which a bilingual individual is placed.

### Language Dominance Moderating the Relationship Between Language Control and Domain-General Control

We predicted that the process of top-down language inhibition involved in speech production would be related to domain-general inhibitory control. Inconsistent with some prior findings in production (e.g., Linck et al., 2012; Liu et al., 2014, 2017) and in comprehension (Litcofsky and Van Hell, 2017; Adler et al., 2020; Bosma and Pablos, 2020), we did not find any overlap in bilinguals between the inhibition-related language switch cost and the non-linguistic Simon congruency effect (as an index of inhibitory control), neither in speed nor accuracy. However, this result corresponds to some other previous findings of no association between linguistic inhibition and domain-general non-linguistic inhibitory control from the perspective of both behavioural (e.g., Calabria et al., 2012; Branzi et al., 2016) and neurocognitive studies (Verhoef et al., 2009; Magezi et al., 2012). It further adds evidence to the proposal that the bilingual practice of linguistic inhibition of the interfering language in the process of speech production is specific to the language domain and that there is limited or no generalisation to non-linguistic inhibitory control measured by conflict resolution tasks (de Bruin et al., 2015; Paap et al., 2015).

However, it would be incorrect to conclude that there is no link whatsoever between domain-specific and domain-general control. To further respond to the second research concern, we correlated the measures of the overall switch costs (irrespective of language), the global responses across trial types in the language task with the non-linguistic measures of both the Simon effect and conflict monitoring indexed by the global reaction times (or accuracy rates) in the Simon task. Consistent with prior studies of bilingual advantage mostly manifesting on monitoring (e.g., Costa et al., 2009; Hilchey and Klein, 2011; Bialystok et al., 2012), it is found that all bilinguals, independent of their language dominance, show an association between the globally sustained language control in the production-based mixing language tasks and global performance in the Simon task in terms of response speed. The current result, i.e., the overlap between two equivalent measures of sustained monitoring control across domains, lends some support to the proposal by Struys et al. (2019b) that conflict monitoring contributes to the language switching process. A domain-general account of monitoring was initially proposed for bilingual language comprehension, but the present results suggest that it can now be extended to include language production as well. Following this monitoring account, the continuous assessment of the possibility of an upcoming language switch in the picture naming task is comparable to the proactive evaluation of the chances of a subsequent occurrence of the stimulus-response conflict in the cognitive task. Our findings suggest that independent of language dominance, bilinguals proactively manage the activation level of the competing languages in language switching, and this monitoring control can be primarily used to assess the possibility of a subsequent language switch.

Additionally, separate group analyses for L1- and L2-dominant bilinguals were conducted to find out if dominance could have an impact on the relationship between language control and domain-general control. It was found that both L1- and L2-dominant bilinguals exploited sustained monitoring control in the overall language switching process, while for L1-dominant bilinguals specifically, it was found that faster global naming response latencies were associated with a smaller size of the Simon effect. This indicates that bilinguals with more L1-dominance tend to exploit domain-general inhibitory control to alleviate the sustained language control in the process of managing the unpredictably upcoming language. However, bilinguals with more L2-dominance exclusively recruit monitoring control to facilitate the overall performance in the language switching process. These results reveal, for the first time, that language dominance moderates the relationship between domain-specific and domain-general control. It suggests that the exploitation of underlying cognitive mechanisms differentiates between L1-dominant and L2-dominant bilinguals. One possible account for the finding is that bilingual individuals with varying language dominance patterns engage in different types of code-switching in the Uyghur-Chinese bilingual community, and only those executive functions that are needed to underlie these language practices are impacted. It may be that L1-dominant bilinguals require an effortful suppression of the dominant L1 when they are exposed to the single L2 sociolinguistic context (Hofweber et al., 2020), and this may lead to an improvement in domain-general inhibitory control. However, this is not a full explanation for our finding, because it is known from previous studies that their possibly exclusive language practice of insertional code-switching only recruits a medium level of inhibition, which is lower than what is needed during alternational code-switching (Treffers-Daller, 2009). Bilinguals who are more proficient in the L2 (Chinese) majority language would be able to switch more complex constituents, such as switches of verbal compounds (mixed Uyghur verbs), and engage in alternation (Backus, 1996). Even though alternational code-switching is supposed to recruit strong inhibition, according to Treffers-Daller (2009), our findings showed that monitoring control is specifically employed by L2-dominant Uyghur-Chinese bilinguals, which may be related to individual variation of L2-dominant bilinguals in selecting the strategies for code-switching. L2-dominant bilinguals in the Uyghur-Chinese bilingual community might engage more in the switching of verbal compounds and less in alternation. Switches of verbal compounds involving items from both languages inserted into a shared grammatical frame may trigger linguistic co-activation and require high monitoring control, even though it should be admitted that whether or not switches of verbal compounds constitute dense code-switching is difficult to define due to the lack of research into this phenomenon in the Uyghur-Chinese bilingual community.

Nevertheless, the design of the present study did not provide further information on bilingual individual differences in code-switching behaviour, and this is one of its limitations. For future studies on bilinguals in a minority/majority language sociolinguistic context, we recommend examining the modulating role of the code-switching type in language control and cognitive control. This can be tested using the methodology of self-reporting code-switching *via* questionnaires (Soveri et al., 2011; Hofweber et al., 2016). We predict that bilinguals who more frequently engage in dense code-switching or switches of complex constituents from both languages will recruit monitoring control to facilitate language switching by reducing mixing costs or asymmetry in switch costs.

## Conclusion

We observed a novel pattern of asymmetry switch costs in more L2-dominant bilinguals within a minority/majority language context who showed a larger switching effect in their L2 rather than in their L1. Our findings suggest that exposure length to the L1 minority language and the age of L2 acquisition affect language control in the process of production, which lends further support to the adaptive control model. Moreover, we found no relationship between inhibitory control processes during the language production task and similar inhibitory domain-general processes in a Simon task. However, our findings lend some support to a domain-general monitoring account of bilingual language production, in that sustained monitoring control was exploited in global language control for both more L1- and more L2-dominant bilinguals. Additionally, more L1-dominant bilinguals showed an overlap between non-equivalent measures of global language control and cognitive inhibitory control. We suggest that language dominance may exert a moderating role in cross-domain generality and that bilinguals with a high frequency in switching practice correspondingly strengthen domain-general conflict monitoring control.

## Data Availability Statement

The raw data supporting the conclusions of this article will be made available by the authors, without undue reservation.

## Ethics Statement

The studies involving human participants were reviewed and approved by the Academic Committee of Shaanxi Normal University. The patients/participants provided their written informed consent to participate in this study.

## Author Contributions

RW: conceptualisation of research, methodology of experiment tasks, collection of data, statistical data analyses, and the draft and revision of manuscript. ES: conceptualisation of research and critical revisions as well as editing on all phases. All authors contributed to the article and approved the submitted version.

## Conflict of Interest

The authors declare that the research was conducted in the absence of any commercial or financial relationships that could be construed as a potential conflict of interest.

## Publisher’s Note

All claims expressed in this article are solely those of the authors and do not necessarily represent those of their affiliated organizations, or those of the publisher, the editors and the reviewers. Any product that may be evaluated in this article, or claim that may be made by its manufacturer, is not guaranteed or endorsed by the publisher.
